# A hybrid GAN–SegFormer–YOLOv9 framework with fuzzy logic for pothole detection and severity classification

**DOI:** 10.1038/s41598-026-48201-2

**Published:** 2026-04-17

**Authors:** R. Kothai, N. Prabakaran

**Affiliations:** School of Computer Science and Engineering, Vellore Institute of Technology, Vellore, 632014 Tamil Nadu India

**Keywords:** Engineering, Mathematics and computing

## Abstract

Pothole detection has become an important task for any country to safeguard its people and transportation from being damaged on road surface which in return help the country to reduce the number of accidents and improve economic growth as well. Deep learning networks has been effective over the years and yielded trustable results for the researchers in attaining accuracy of detection and its severity classifications. General Adversarial Networks are efficient in improving the results based on the discriminator feedback that helps to come out with enhanced performance of pothole detection. In this paper, we propose a GAN with Relativistic Discriminator based image enhancement neural network with SegFormer-B4 integrated with Transfer Learning, finally YOLOv9 is incorporated with Fuzzy Rules to determine the severity of potholes based on diameter and depth parameters of the founded bounding boxes. The severity classifications are categorized into low, medium and high, based on the detected severity category the required recommendations are determined as crack sealing, patching work and patching and reconstruction. The proposed model was implemented in MATLAB2021a simulation environment using datasets such as Japan, POTHOLE V3 and Pothole Detection Dataset-V2-2022 and results were compared with the other approaches like YOLOv7, YOLOv8, Faster-RCNN and CC-ViT approaches in terms of accuracy, precision, F1-score, mAP@0.5 and recall performance evaluation metrics.

## Introduction

The quality road infrastructure has become very crucial for every country as it provide major contribution for social interaction, transportation of goods to improve economic activities and also public safety as well. But, the durability and integrity of roadways and its surfaces are heavily disturbed by severe weather conditions, natural disasters and over traffic conditions with heavy loads that leads to the structural damage of the road surface such as cracks and potholes which possible produce endangers to the road users and also increase the cost of road maintenance for the respective authority^1,2^. Potholes are considered to have high impact on road maintenance cost and also damage vehicles tire longevity due to uneven depression of its structure due to water infiltration and vehicle pressure. In addition, these defects on roads are widespread, thus pose significant risks for motorists and other road users as well^3^. Therefore, it is important to ensure both vehicle and road users safety by effectively detecting potholes on roadways. Major contributors of potholes are climate change and low-standard materials used by few construction companies^4,5^. Detection potholes in early stages would help ensuring the driver safety and reduce accidents, vehicle damage and unpleasant driving experiences. Basically, the pothole detection is manually performed by structural engineers and certified inspectors through Manual Visual Inspection (MVI). When these potholes are not repaired in timely manner, the weather change and load carried by the vehicles will further increase pothole size in terms of depth and diameter wise, thus road users will be seriously affected. Though, manual detection of potholes were adopted by most of the countries like China, UK, USA, India and more, the process seems to be difficult, tedious and inefficient. In rainy seasons, detecting the potholes is very hard as it will be covered by water, typically its size can get expanded due to rain and transportation loads. Potholes can bring damage to typical four wheelers in the form of dents in the rims, deflated tyres, and change in wheel alignment, suspension components and more^6–8^. To overcome the disadvantages of MVI, Digital Inspection Vehicle (DSV) has been used by many agencies across the globe to collect data about potholes presence, yet it needs to be processed to extract potholes size, depth, length and severity features. Hence, DSV is expensive; such data survey is conducted either annually or bi-annually. Following this, cameras were found to be the common to capture images of potholes due to its low cost availability, easy interpretability of image capturing and applying AI-based object detection on the captured image. Camera captured images are not always contain enough information about potholes, especially estimating the dimension of the potholes are very challenging^9–11^. So, some studies have employed Light Detection and a Ranging (LiDAR) sensor which is built to measure the distance to the potholes or cracks with precision using the inbuilt laser lights on these sensors and this has widely been used in traffic monitoring and autonomous driving systems that has reduced the required production cost and also improved the assessment of pavement conditions on road surface^12,13^. Data collection is one the most preliminary step and vital using automated vehicle equipped with cameras and laser imaging systems for both 2D and 3D imaging of road pavement surfaces in complex environment employing various methods and hardware like Ground Penetrating Radar (GPR), Falling Weight Deflecto-meters (FWD). Following data collection, the collected information needs to be processed by adapting different deep learning models and algorithms to identify distress on surface, damages, roughness of pavement and skid resistance. Finally, developing a data analysis model to accurately identify the cracks and potholes to ensure traffic safety and driving comfort. However, the present distress algorithms with traditional and machine leaning approaches resulted in poor recognition rate of potholes and its severity, especially under complex environmental conditions. Yet, deep leaning methods can establish a multi-layer neural network for abstracting complex information by learning distress presence from large amount of data to ensure accurate detection of potholes^14–17^. You Look Only Once (YOLO) models is the most-widely used object detection algorithm which makes it more suitable for identify potholes from static data images and videos, especially versions like YOLOv5, YOLOv7 and YOLOv8 have proved to be effective and accurate when trained on custom datasets for learning potholes in various road conditions. Despite significant progress in automated pothole detection, existing approaches predominantly rely on either deep learning-based classification or loosely coupled combinations of segmentation, detection, and rule-based decision models. Such approaches tend to process the image enhancement, feature extraction and severity assessment as separate steps and prevent their effectiveness in the face of variability and uncertainty on the real world. In order to overcome these shortcomings, this paper offers a measurement-based, end-to-end hybrid model, which closely connects relativistic GAN-based task-aware image enhancement, transformer-based metric-aware semantic segmentation, geometry parameter extraction using YOLOv9, and uncertainty-aware severity inference, founded on Mamdani fuzzy logic. In contrast to more traditional hybrid systems that utilize fuzzy logic over heuristic severity indicators or confidence scores, the suggested system utilises directly on the physically measured pothole characteristics, i.e. diameter and depth, of coordinated segmentation and detection results. This unified design transforms pothole analysis from a categorical detection problem into a quantitative decision-support framework, enabling reliable severity assessment and actionable maintenance recommendations under diverse imaging conditions.

**Figure 1:**
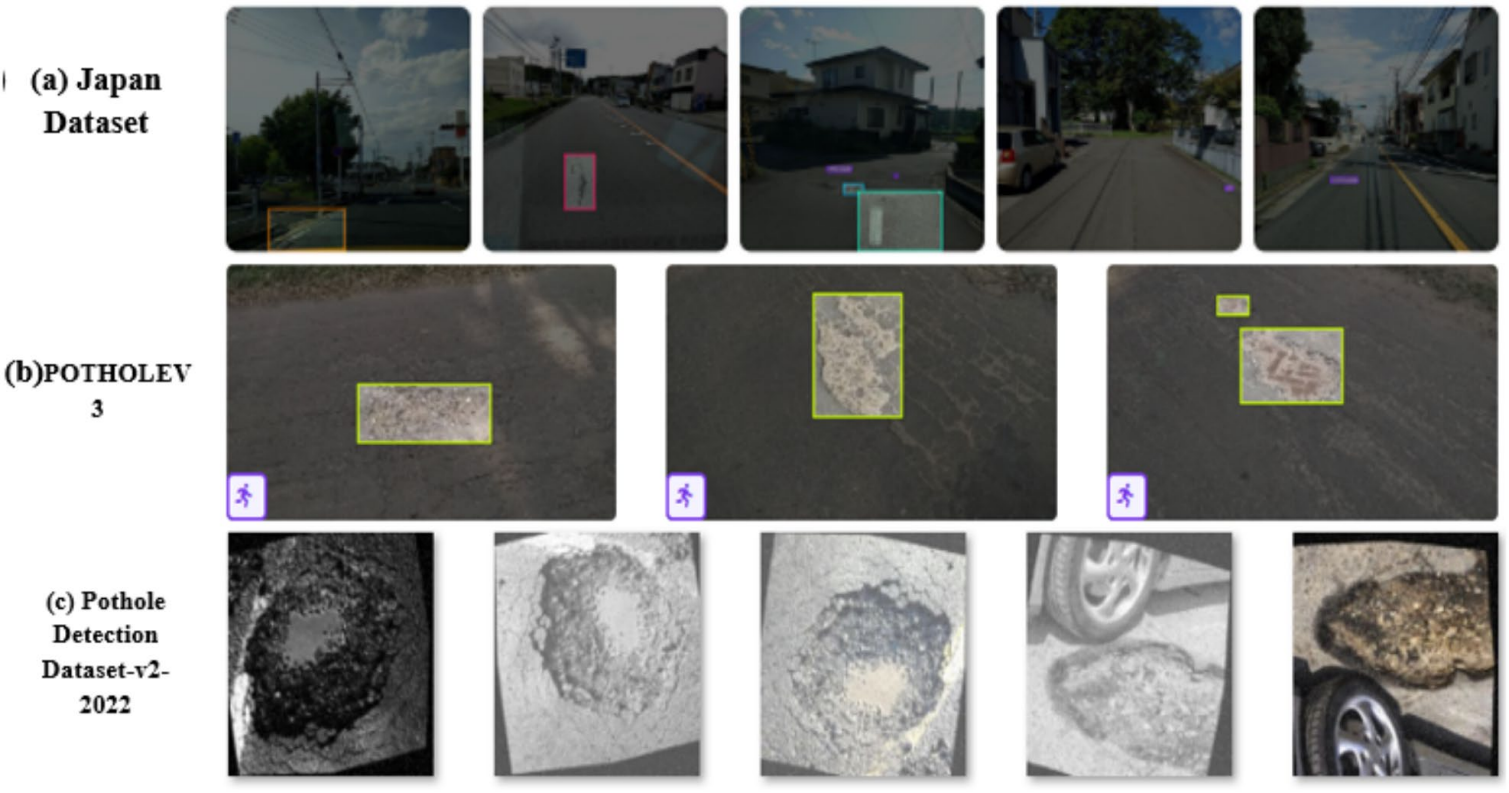
Sample image from each dataset.

The contributions of this research work are:

- General Adversarial Networks with Realistic discriminator is used for image enhancement purpose with data augmentation methods for enhancing the datasets image quality and resolution.
- SegFormer-B4 with Transfer Learning is adapted for training the model and to identify potholes on the road surface by delineating the exact boundaries of potholes by drawing bounding boxes.
- The output from the SegFormer4 is used to measure the quantitative measurement of potholes like diameter and depth.
- YOLOv9 with fuzzy rules is applied (if...then) on those quantitative measurements to determine the severity of detected potholes on the basis of fuzzy rules.
- Based on the severity category of low, medium and high, the recommendations are given for the road maintenance authorities.
- The novelty of this research is the integration of SegFormer 4 and Yolov9 with Fuzzy based Rules for decision support in post-processing, which is vital in ensuring the enhancement in pothole detection accuracy with high positive detection since the approach has adopted GAN model performs image enhancement/data augmentation and also improves the performance as the model is trained and tested repeatedly on different datasets from various regions and continents.

**Figure 2:**
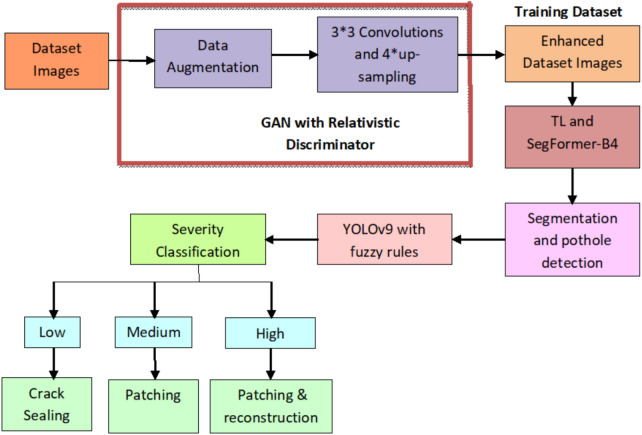
Overall architecture pipeline of the proposed approach.

## Literature work

An architecture utilizing transfer learning with a Segformer network was proposed^18^ which employs Intel RealSense D435i camera to generate dataset with a total of 583 images out of which 299 images consists of potholes and the rest consists of environmental noise on various road pavement surfaces. The proposed network model was evaluated using different metrics like accuracy (86.8%), recall (90.87%), F1 Score (90.433%), precision (90.01%) and a loss (0.0431%). The model has also achieved accurate diameter estimation of potholes using IOU of 85.872% and depth estimation error of 5.94mm. Fuzzy logic system was used to process these estimated measurements to recommend repair urgency with appropriate repair techniques. To address the challenges in detecting water filled potholes and those shaded by trees, a novel approach using cascade classifier incorporated with vision transformer^19,20^ was proposed in which the classifier identifies the patterns and transformer performs in depth analysis and classification. The proposed models was trained under various datasets like ICTS, GTSRDB, KAGGLE, CCSAD and the performance was compared with YOLOv3, YOLOv4, Faster RCNN and SSD. The results showed that the proposed model achieved improved performance with mAP of 97.14% for traffic sign detection and 98.27% for pothole detection. A Segformer based framework^21^ was proposed for pothole segmentation on high resolution RGB images captured by a road inspection vehicle and the model doesn’t require explicit decoding paths which results in accurate segmentation process. The captured dataset contains 600 images using ZED stereo camera at 400*400 pixels and the dataset images were composed with the ratio of 240-180-180 for train, validation and test purposes. The Segformer (b5) model performance was compared with U-Net (S5-D-16) model in terms F1 score. To manage the class imbalance, a dice loss function was used to set weights of 80%-20% for potholes and environment noises. The proposed model has achieved average accuracy of 96.21%, average precision of 83.07%, Average recall of 72.17%, average F1 score of 77.24% and average IoU of 62.91% whereas U-Net has resulted in 91.34%, 51.95%, 66.78%, 58.44% and 41.28% respectively. An effective pothole surveillance system^22^ utilizing LiDAR combined with Global Navigation Satellite Systems (GNSS) receiver to identify the localization of potholes was proposed with four states: first, a ring-wise cross sectional image was generated after processing LiDAR data. Second, the presence of potholes with its size is predicted using deep learning object detection network. Third, aggregate the ring-wise inferences to make final decision. Finally, generate inspection map by synchronizing aggregated inferences with GNSS. The proposed model has achieved 100% accuracy on the datasets with the error rate of potholes in terms of depth, width and length was at 0.27, 2.58 and 5.15 inches respectively at the speed of 55 mph. at the speed of 65mph, the error was 0.47, 1.87 and 7.23 inches respectively. The SPFPN-YOLOv4 tiny^23^ was modeled by integrating Spatial Pyramid Pooling and feature pyramid network with CSPDarknet53-tiny. A total of 2665 dataset images were obtained and undergone data augmentation process, then composed into 70%, 20% and 10% for training, validation and testing. The proposed model performance was compared with models like YOLOv2, YOLOv3, YOLOv4 where the proposed SPFPN-YOLOv4-tiny showed the performance improvement off 2%-5% on mAP@0.5. The distance estimation equation and pinhole camera on tire contact patch size and the pothole size. Using YOLOv7 machine learning technique^24^, a pothole detection system was proposed to warn the drivers about the presence of potholes on road in prior with a buzzer sound while approaching potholes on road. The model was implemented with high quality GPU processor with Googlecolab machine learning tool sing python language. The datasets were taken from kaggle with the composition of 1265, 401 and 118 for training, validation and testing respectively. The model performance was compared with CNN model where YOLOv7 achieved 94.5% accuracy by processing 45 frames per second. Road-TransTrack^25^, a tracking model using transformer optimization was proposed with YOLOv5 classification network which used dataset images consists of potholes and cracks. The model was enhanced using self-attention mechanism and transformer, finally the trained model was tested on videos to very its effectiveness where the model achieved 91.60% and 98.59% accuracy in detecting potholes and cracks, 0.9847 and 0.9417 of F1-score respectively. Achieving precise segmentation is always a challenging task due to the complex image backgrounds; to address this issue the study collected different potholes of three categories, namely, normal, fully damaged and severely damaged with a total of 2,097 images. A segmentation architecture^26^ using Atrous Spatial Pyramid Fusion (ASPF) and Channel Attention Module (CBAM) where ConvNext placed as backbone to extract multi-depth features, then integrated with ASPF which captures multi-scale contextual information. Finally, CBAM module was embedded in between the above two modules for adaptive channel and spatial feature calibration. An ablation study was carried out where the full model has achieved a better performance.

## Problem description and motivation

The high rate of urbanization has caused very high traffic congestion in urban centers, which puts a lot of pressure on the standards of road networks. Small lanes, poor signs and poor road networks like potholes have severe safety hazards to the road users and their vehicles. Proper and timely pothole identification is thus a need in good road maintenance and prevention of accidents. Despite the fact that a number of pothole detection models based on deep learning have been suggested, their performance is usually constrained by noise in images, changes in resolution, and adverse real-life lighting situations. Other recent work has tried to enhance the accuracy of detection by combining deep learning with LiDAR sensors but LiDAR systems are currently very expensive and therefore can not be practically deployed in large scale road monitoring systems. Although potholes can be considered anomalies due to their deviation from normal road surfaces, the availability of labeled datasets in this study enables the problem to be more appropriately formulated as a supervised detection and classification task rather than a purely unsupervised anomaly detection problem. Accordingly, this work models pothole identification as a hybrid detection–segmentation–severity classification problem, where deep learning techniques are employed to accurately localize and characterize road surface defects. This formulation ensures consistency between the dataset characteristics and the adopted methodology, aligning with standard practices in computer vision-based road infrastructure monitoring. Motivated by these challenges, this study focuses on pothole detection using only static RGB images captured under diverse environmental and lighting conditions. The primary objective is to enhance pothole detection accuracy through an efficient deep learning framework with robust training, testing, and validation strategies, Furthermore, the use of multiple real-world datasets from different geographical regions introduces variability in road conditions and illumination, enhancing the robustness and generalization capability of the proposed model. However, large-scale real-time deployment and validation across broader environments remain important directions for future work.

## Proposed work

### Model description

In this paper, we propose a GAN based image enhancement with data augmentation network model to enhance the image quality of the dataset images integrated with pre-trained SegFormer-B4 transformer for synthetic segmentation of images to extract high-level features in order to detect potholes in images. The GAN module operates as a pre-processing stage, where a generator network composed of stacked 3$\times$3 convolutional layers and multi-stage up-sampling enhances contrast, edge sharpness, and surface texture, while a relativistic discriminator ensures perceptual realism during adversarial training as illustrated in Fig. 2. Then, the transformer is fine-tuned using transfer learning for quick learning of potholes characteristics in the undertaken dataset images. The GAN-enhanced images are used exclusively during the training phase to improve robustness under challenging conditions such as low illumination, shadows, and surface degradation, while original images are retained during validation and testing to ensure unbiased evaluation. The augmentation details are presented in the dataset description section. Once the low-quality images are enhanced in terms of quality and resolution, SegFormer-B4 effectively performs semantic segmentation, enabling accurate identification of pothole regions through improved feature representation. The segmentation outputs are subsequently forwarded to the detection stage, where YOLOv9 integrated with fuzzy rule-based inference processes the spatial features for precise pothole localization. With fuzzy rules adoption along YOLOv9, the severity levels of identified potholes are determined and recommended actions are suggested.

**Figure 3:**
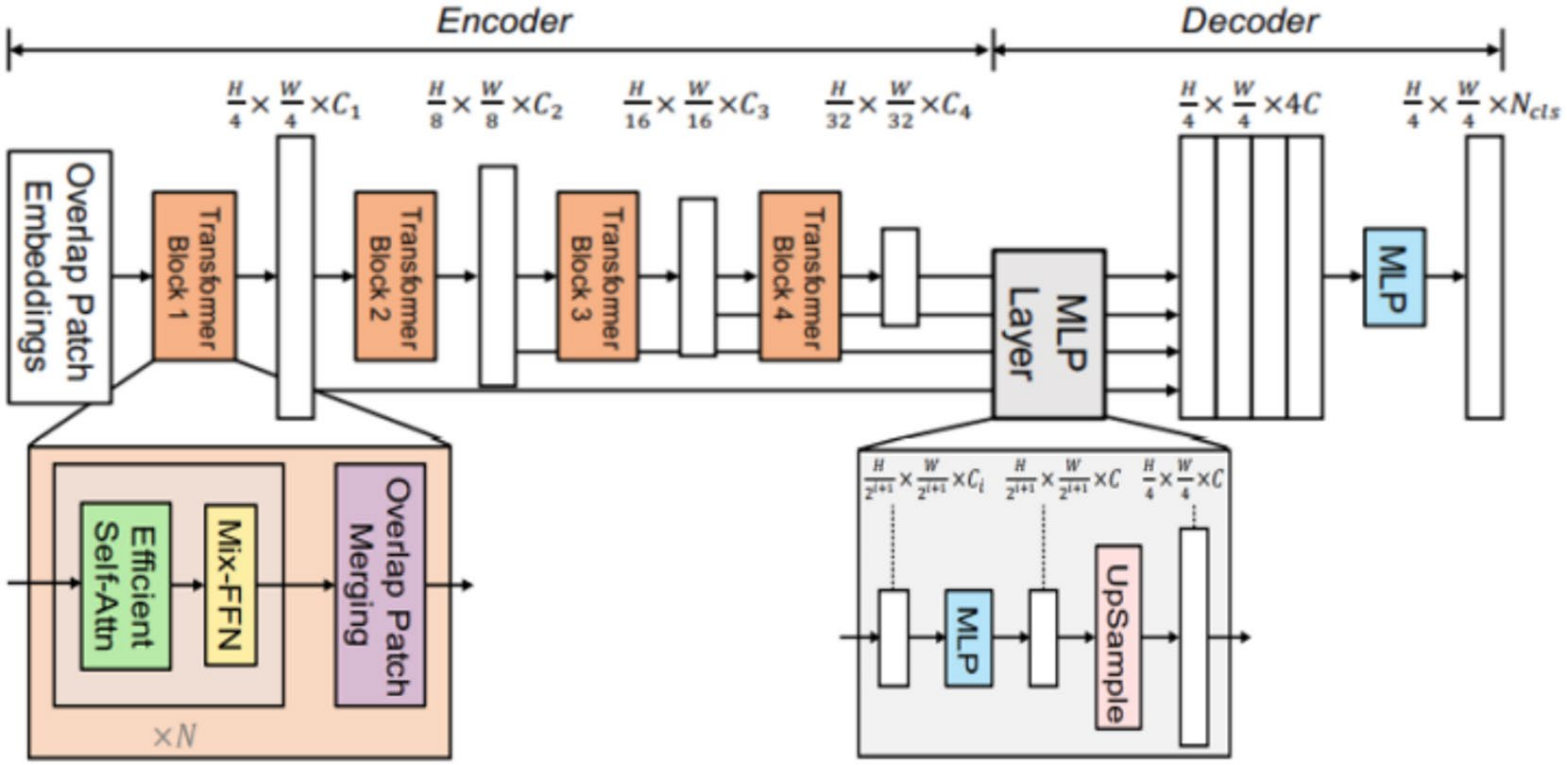
SegFormer architecture.

### General adversarial networks

Figure 2 illustrates the overall architecture pipeline of the proposed approach, highlighting the role of the GAN with a relativistic discriminator in the image enhancement stage and its integration with downstream segmentation and detection modules. As shown in Fig. 2, the input dataset images are first passed through a data augmentation block, where geometric and photometric transformations such as rotation, flipping, cropping, and scaling are applied. These augmented images are then fed into the generator network of the GAN, which consists of multiple 3$\times$3 convolutional layers followed by four up-sampling stages, as indicated in the figure. This design enables progressive restoration of spatial resolution while enhancing contrast, texture details, and edge clarity in degraded road surface images. The GAN employs a relativistic discriminator, replacing the conventional discriminator to improve training stability and perceptual quality. Instead of independently classifying images as real or fake, the relativistic discriminator evaluates whether a real image appears more realistic than a generated one. This formulation is particularly effective in preserving fine-grained pothole boundaries and surface irregularities, which are critical for accurate segmentation and detection.

#### GAN training configuration and hyperparameters

The GAN is trained using paired original and enhanced images with a combined relativistic adversarial loss and L1 reconstruction loss, ensuring both perceptual realism and structural fidelity. The generator and discriminator are optimized using the Adam optimizer with a learning rate of 0.0002, momentum parameters $\beta _1=0.5$ and $\beta _2=0.999$, and a batch size of 16. The network is trained for 200 epochs with an alternating update strategy (1:1 generator-to-discriminator update ratio). Weight initialization follows a normal distribution with zero mean and standard deviation of 0.02. Once trained, only the generator is retained to produce enhanced dataset images, as shown in the “Enhanced Dataset Images” block in Fig. 2. The discriminator is discarded after training. Importantly, GAN-based enhancement is applied only to training images, ensuring no data leakage during validation or testing.

### Segformer with training learning

The determination of potholes in an image was identified by applying transfer learning in segformer transformer since it identifies the object of interest with a semantic segmentation. In the initial stage, we performed data augmentation on the selected datasets which consists of thousands of images with potholes. After the segmentation, we compute the diameter and depth of these potholes using fuzzy rules integrated with YOLOv9. A pre-trained Segformer^27^ on the considered datasets, following this transfer learning is applied to take the advantage of its reuse feature. The Segformer-B4 is built with components of hierarchical transformer encoder and a lightweight all MLP decode head. At first, hierarchical transformer is trained on the datasets before adding a decode head without fine tuning. We have adapted Segformer-B4 transformer instead of other transformers due to the fact that it does not use positional encoding as it could lead to worse test-time performance especially when the inference resolution is not same as the training image resolution. Moreover, the computational overhead of MLP is negligible. The Segformer-B4 architecture has been presented in Fig. 3. With encoder, decoder and attention module, the segformer can focus on important local and global features of an image where potholes are detected. The datasets used in this study was constructed to enhance the model’s performance in accurately detecting potholes and classifying its severity in different scenarios and environments. Though, segformer-B4 being the slowest than other transformers accuracy achieved is best among all other transformers. Each transformer block is embedded with Multi-head-attention blocks, Feed Forward Blocks and Patch Merging Blocks where the decoder is made of Linear and up-sampling layers.

###  YOLOv9 integration with fuzzy model

**Figure 4:**
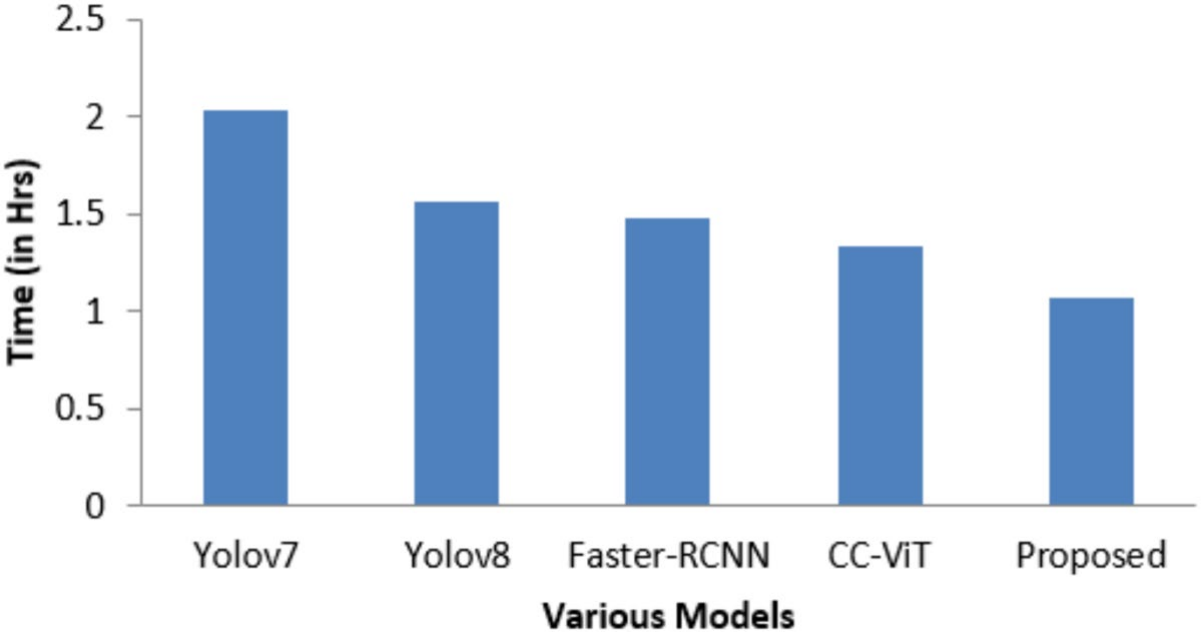
Comparison of training time on models.

**Figure 5:**
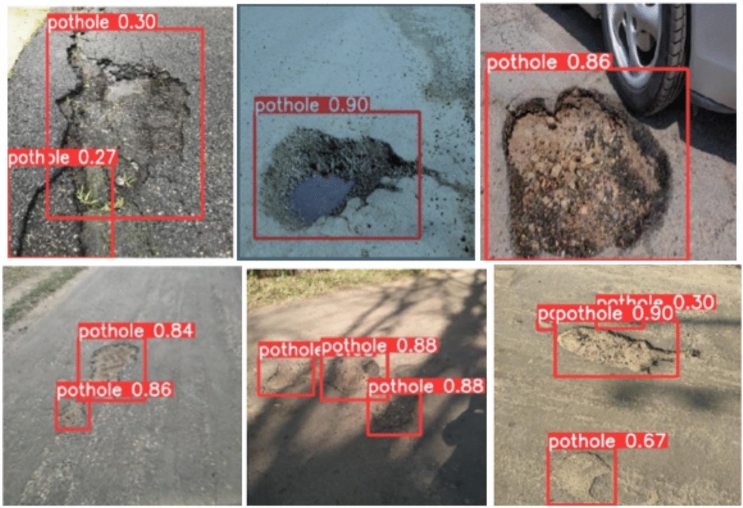
Sample dataset images of predicting potholes depth.

We have considered Yolov9 for this study due to its faster inference speed and light-weight architecture with the advancements of Programmable Gradient Information (PGI) GELAN which optimizes gradient propagation and feature aggregation with reduced parameters and computation without sacrificing accuracy. When a fuzzy model is integrated with YOLO models, it tends to produce better results in terms of pothole detection accuracy. Here, we propose the integration of a fuzzy model with YOLOv9 to determine the severity of potholes by effectively quantifying damage using key parameters such as depth and diameter. The severity levels are categorized into Low, Medium, and High, which support repair urgency decision-making. The bounding box parameters, particularly depth and diameter, are used where depth determines repair urgency and diameter supports maintenance recommendations.

For each detected pothole, geometric parameters are extracted from the bounding box and segmented region, namely diameter and depth. The diameter is estimated using the projected pixel radius and horizontal scale calibration as:

1 $$\begin{aligned} P D r_{m s}=R o_{p s} * H Z_{\text{ scale } } \end{aligned}$$

where $R o_{ps}$ represents the radius of the segmented pothole in pixel space and $HZ_{scale}$ denotes the pixel-to-meter horizontal conversion factor obtained through camera calibration.

2 $$\begin{aligned} \text{ Pothole } _{\text{ dth } }=\operatorname {DoP}_{S F E}-\operatorname {DoP}_{D P} \end{aligned}$$

where $DoP_{SFE}$ corresponds to the distance from the camera to the surrounding flat road surface and $DoP_{DP}$ represents the distance to the deepest point of the pothole. These distances are obtained from the depth estimation map aligned with the segmented pothole region. Prior to depth computation, all images are geometrically corrected using camera intrinsic calibration parameters to minimize projection distortion. Diameter primarily supports maintenance recommendation planning, while depth serves as the primary indicator of structural severity and repair urgency. Due to inherent uncertainty in depth measurements caused by illumination variation, occlusion, and surface irregularities, severity estimation is handled using a Mamdani fuzzy inference engine. The defuzzified pothole depth is computed as:

3 $$\begin{aligned} D o P *=\frac{\sum v C T(D o P) D o P}{\sum v C T(D o P)} \end{aligned}$$

where *vCT*(*DoP*) denotes the membership confidence of each fuzzy rule. The final severity class is determined based on combined fuzzy inference of depth and diameter features. The integration of Mamdani fuzzy inference enhances interpretability by mapping quantitative measurements (diameter and depth) to human-understandable severity levels. Unlike conventional black-box deep learning outputs, this rule-based system provides transparent and explainable decision-making. However, further improvements such as explainable AI (XAI) techniques and user-centric validation are required for large-scale infrastructure deployment. To standardize the data with reduced impact differences as far as the scale and magnitude is concerned with respective attributes just to overcome bias on the basis of differences in magnitude, min-max normalization was employed with the following equation,

4 $$\begin{aligned} M M_X=\frac{X-X_{\min }}{X_{\max }-X_{\min }} \end{aligned}$$

This integrated YOLOv9-fuzzy framework ensures robust severity classification by jointly leveraging geometric measurements and uncertainty modeling, thereby improving reliability for real-world road maintenance decision-making.

**Table 1:** Fuzzy model inputs and outputs for severity classification.

Diameter	Depth	Severity	Classification
Low (10–15 cm)	Low (10–20 mm)	Low	Crack Sealing
Medium (15–40 cm)	Medium (20–35 mm)	Medium	Patching Work
High (From 45 cm and above)	High (>= 35mm)	High	Patching and reconstruction

Table 1 presents the different diameter and depth variable numbers to determine the severity level of the detected pothole and the required likely action to be taken for road surface maintenance with good quality to prevent vehicles and road users from damage.

### Novelty and distinction from related hybrid approaches

The recent research has covered the hybrid systems that are based on image segmentation, object detection, and fuzzy logic to examine road damage or potholes. Although these works do illustrate the practicality of multi-stage pipelines, the majority of them combine these elements in a loosely coupled or sequential way with segmentation taking on the role of region identification, detection being reduced to categorical classification and fuzzy logic processing on heuristic designation or confidence rating. There is a basic difference in the way every module is structurally designed and flow of information between the stages is executed in the proposed structure, which leads to a measurement-oriented and decision-based architecture. Unlike existing hybrid models, the proposed framework introduces a measurement-driven integration where segmentation and detection outputs are directly used for quantitative severity estimation, rather than categorical classification. This tightly coupled design distinguishes it from loosely connected hybrid pipelines. The first aspect is that, in contrast to traditional GAN-based methods of preprocessing, which focus on either visual or noise minimization, the relative formulation of the framework uses a relativistic discriminator-based GAN to specifically optimize on preserving boundaries and improving surface discontinuities. This design decision is vital to the pothole analysis since fine-grained boundary information and depth visualization have a direct impact on the accuracy of the segmentation and the accuracy of the geometry measurement that follows. The best we know is that there are no task-aware pothole detection pipelines based on relativistic GANs as a stage of enhancement that uses downstream metric extraction. Second, unlike the previous segmentation-based methods that adopt semantic masks as the localization method, the SegFormer-B4 module in this study works as a metric-aware segmentation phase. The pothole segments are not regarded as the final products but rather they serve to facilitate accurate determination of the physical characteristics, including diameter and depth. This changes the role of segmentation form categorical labeling to the quantitative structural analysis which is highly missing in the prior segmentation-detection hybrid systems. Third, the majority of the literature has been using direct class prediction or classifying severity based on confidence to detect and classify the severity of potholes, whereas object detectors like YOLO have been extensively used in pothole detection and severity classification. Conversely, the proposed framework adopts YOLOv9 as a major geometric parameter extractor, in which the data on the bounding boxes and the segmented regions is shared to obtain the correct diameter and depth. This quantitative application of detection results is what separates the process of the previous YOLO-based classification pipelines. Lastly, as opposed to the current fuzzy logic-based pothole assessment systems, which consider fuzzy inference to heuristic severity levels or subjective rules of the expert, the proposed Mamdani fuzzy inference system uses physically measured and normalized attributes based on the outputs of segmentation and detection systems. The fuzzy module is an uncertainty-sensitive decision support system as opposed to a post-hoc classifier by modeling the uncertainty in measurement of both depth and diameter. This allows maintenance strength measurement and repair advice in the conditions of real image variation. Overall, the novelty of the proposed approach lies not in the isolated use of GANs, transformers, object detectors, or fuzzy logic, but in their tight, measurement-centric integration, where each module is explicitly designed to support downstream quantitative analysis and decision-making. This end-to-end coupling differentiates the proposed framework from existing segmentation–detection–fuzzy pipelines and enhances its suitability for practical road maintenance and infrastructure monitoring applications.

### Model selection stratergy

The selection of the comparative models was on the basis of architectural diversity, real-time feasibility, and equitable benchmarking across pothole datasets. YOLOv7, YOLOv8, and YOLOv9 are subsequent iterations of real-time one-stage detectors, enabling progressive performance assessment. Faster-RCNN is included as a two-stage baseline, and CC-ViT represents transformer-based vision models. Even though more recent models have been developed, many of them are either computationally intensive or lack stable open-source implementations, making them unsuitable for real-time applications. Although models such as Variational Autoencoders (VAE), standard GANs, and reinforcement learning-based approaches have been explored in related domains, they are not widely adopted for real-time pothole detection due to computational complexity and lack of stable implementations for object detection tasks. Instead, this study focuses on widely accepted and reproducible baselines such as YOLOv7, YOLOv8, Faster-RCNN, and CC-ViT, ensuring fair and practical comparison. The selected models provide a balanced trade-off between accuracy, efficiency, and reproducibility. The consistent performance trends observed across datasets further validate the suitability of the selected models and demonstrate the practical applicability of the proposed fuzzy-YOLOv9 framework for real-world road surveillance systems.

## Experimentation results

The proposed model was executed on a windows 8.1 operating system with MATLAB 2021a, installed with the PyTorch framework. The testing was done on a system with a Gpu architecture with a clock rate of 2.7 GHz. SegFormer-B4 was used as the backbone network; it is a powerful network with the ability to extract semantic features of structured and non-structured road surface patterns. The behavior of the proposed framework was analyzed against YOLOv7, YOLOv8, Faster-RCNN, and CC-ViT models to have fair comparison of the benchmark between CNN-based, two-stage, and transformer-based detection architectures. The comparative analysis was conducted based on the standard measures of performance such as Accuracy, Precision, Recall and mAP@0.5.

**Table 2:** Comparative analysis of various metrics on Japan dataset.

Models	Precision	Recall	mAP@0.5%	Accuracy	F1-Score
Yolov7	93.12	92.14	92.87	94.72	92.51
Yolov8	94.54	93.76	94.21	95.12	93.96
Faster-RCNN	94.87	93.12	94.89	95.28	95.12
CC-ViT	95.87	95.14	96.34	96.85	96.83
**Proposed**	**97.84**	**96.21**	**97.82**	**98.92**	**98.14**

**Table 3:** Comparative analysis of various metrics on POTHOLEV3 dataset.

Models	Precision	Recall	mAP @0.5%	Accuracy	F1 -score
Yolov7	93.84	91.28	90.89	91.67	92.19
Yolov8	94.96	94.52	93.65	94.98	93.24
Faster-RCNN	95.12	95.47	95.33	96.37	95.87
CC-ViT	96.84	96.13	97.04	97.54	97.35
**Proposed**	**98.58**	**97.21**	**98.59**	**98.28**	**98.41**

**Table 4:** Comparative analysis of various metrics on pothole detection dataset-v2-2022 dataset.

Models	Precision	Recall	mAP@0.5%	Accuracy	F1 -score
Yolov7	94.05	91.61	92.31	93.13	92.56
Yolov8	95.17	93.1	93.81	95.06	94.12
Faster-RCNN	96.54	95.38	94.83	95.87	95.81
CC-ViT	97.12	96.03	96.54	97.12	96.76
**Proposed**	**98.64**	**97.86**	**98.18**	**98.79**	**97.42**

**Figure 6:**
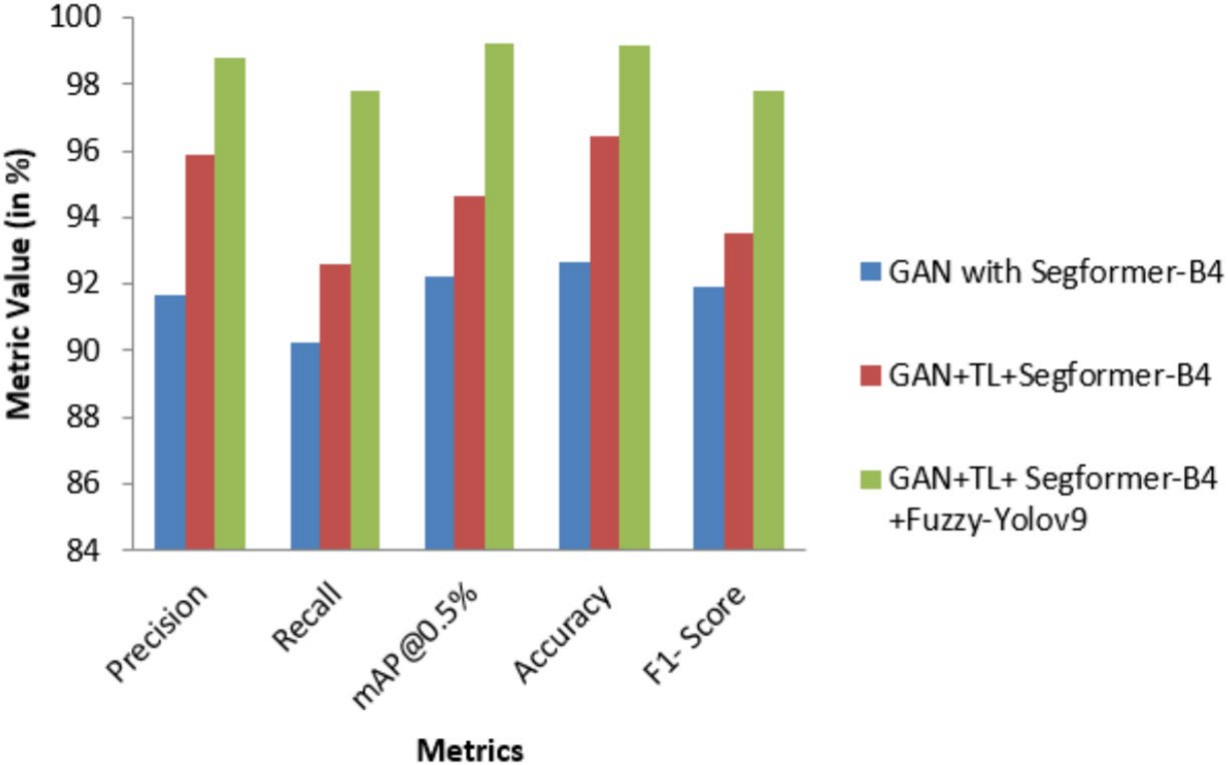
Ablation study observation.

**Figure 7:**
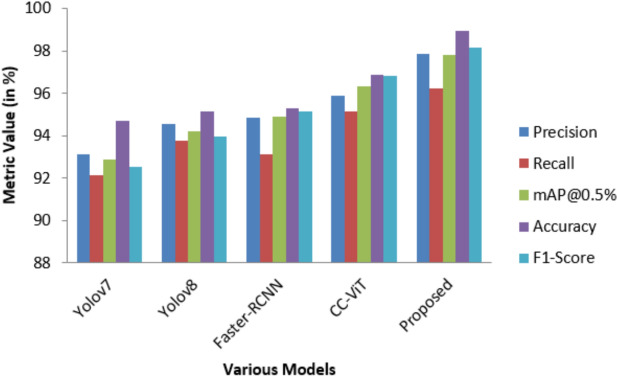
Comparative analysis of various metrics on Japan dataset.

**Figure 8:**
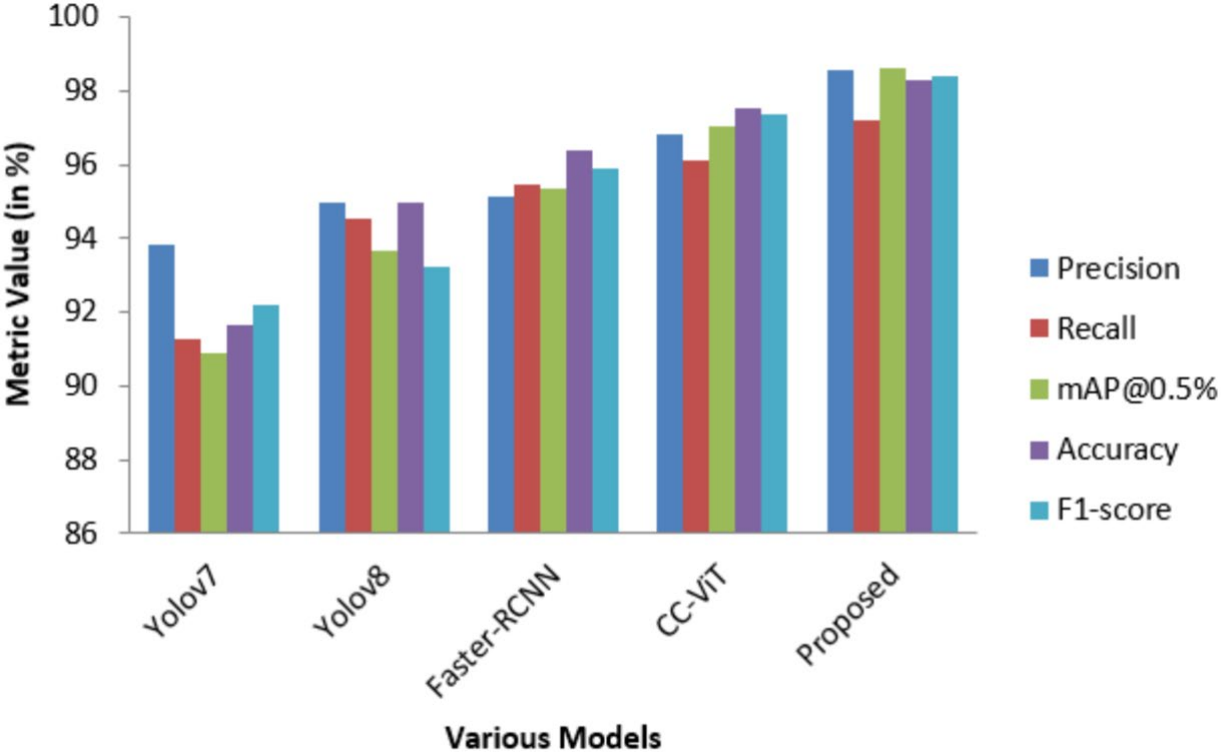
Comparative analysis of various metrics on POTHOLEV3 dataset.

**Figure 9:**
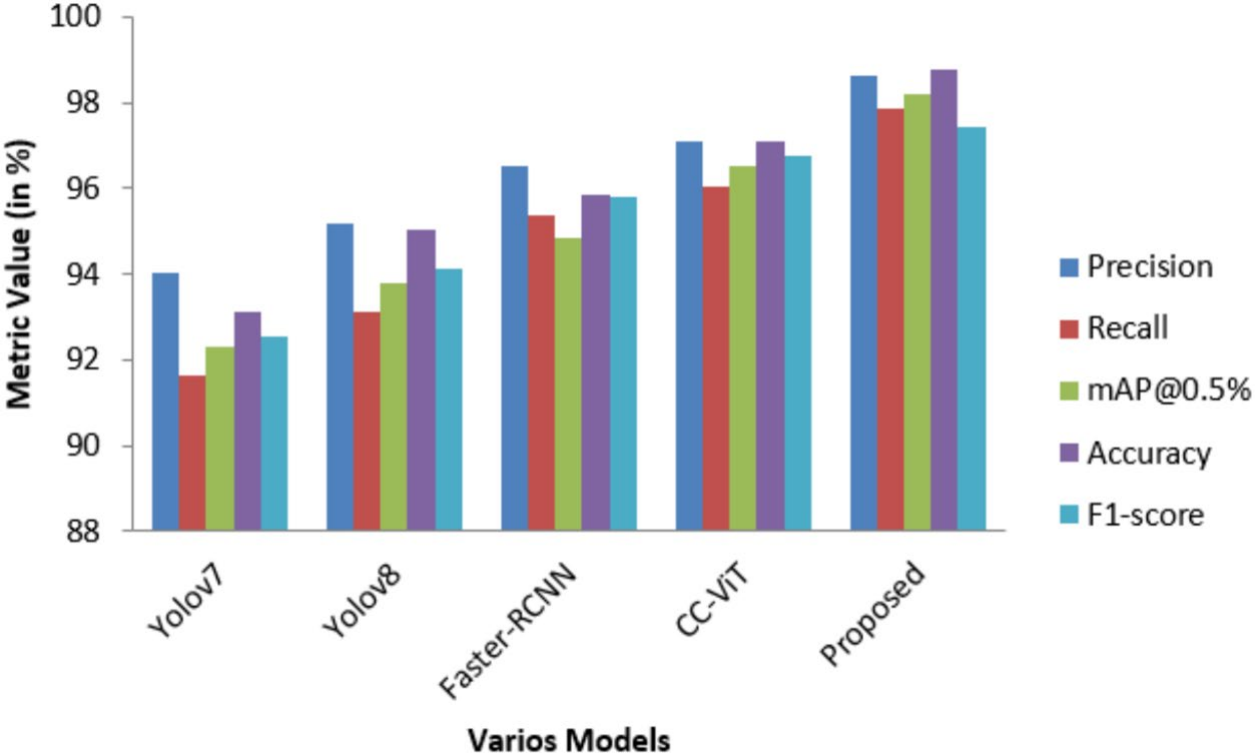
Comparative analysis of various metrics on pothole detection dataset-v2-2022 dataset.

### Dataset

The datasets employed in this study consist of road surface images captured under diverse real-world conditions, including variations in illumination, background texture, camera viewpoint, pavement material, and weather. To ensure geographical and environmental diversity, the images were collected from multiple regions, primarily Japan and China, which differ significantly in road construction standards and surface characteristics. Such diversity is essential for improving the generalizability and robustness of pothole detection models. All datasets used in this work are publicly available, well-curated, and widely adopted in recent computer vision studies on road damage detection. The dataset sources are hosted on Roboflow Universe and Kaggle, ensuring transparency, reproducibility, and fair benchmarking. Although the dataset exhibits a higher proportion of pothole instances, several strategies were adopted to mitigate potential bias. First, stratified data splitting ensured balanced representation across training, validation, and testing sets. Second, multiple evaluation metrics such as precision, recall, F1-score, and mAP were used instead of relying solely on accuracy, thereby avoiding misleading performance interpretation. Furthermore, the use of multiple datasets (Japan, POTHOLEV3, and PD-v2-2022) introduces variability in road conditions, reducing dataset-specific bias. The consistent performance across all datasets demonstrates that the model is not overfitted to a single data distribution.

#### Japan dataset

The Japan dataset was obtained from the Potholes Japan Computer Vision Project hosted on Roboflow Universe. The original dataset comprises 7,404 annotated images covering multiple road damage categories. From this collection, 974 pothole-specific images were carefully selected to maintain class relevance and distribution integrity. All images were resized to 640 $\times$ 640 pixels to ensure consistency during model training. No data augmentation was applied in order to preserve the original data distribution and real-world characteristics, which is particularly important for safety-critical applications such as road infrastructure monitoring^28^

#### POTHOLEV3

The POTHOLEV3 dataset consists of 6,783 road surface images collected under a wide range of environmental and acquisition conditions, primarily from Chinese road networks. The dataset exhibits substantial intra-class variability in pothole appearance, size, shape, and severity, making it suitable for learning robust visual representations. To enhance resistance to environmental variations and mitigate overfitting, controlled data augmentation strategies were applied, including horizontal and vertical flipping, shear transformations ($\pm 12$°and $\pm 13$°), grayscale conversion applied to 25% of the images, saturation variation (-29% to +29%), exposure adjustment ( -9% to +9%) and Gaussian blurring up to 1.75 pixels. These augmentations simulate real-world conditions such as illumination changes, camera motion, and surface degradation. All images were standardized to a spatial resolution of 640 $\times$ 640 pixels. These transformations improve robustness to lighting variations and surface texture changes^29^.

#### Pothole detection dataset-v2-2022

This dataset was collected in Sihao, China, using vehicle-mounted cameras in accident-prone regions. It contains 2,105 images, with one or more potholes per image. Images were resized to 640 $\times$ 640 pixels after EXIF orientation correction. Data augmentation included salt-and-pepper noise addition, cropping (0–20%), horizontal and vertical flipping (50%), random rotation ($-15 ^{\circ }$ to 15$^{\circ }$), brightness and exposure variation (− 25% to +25%), and Gaussian blurring (0–5.25 pixels) to enhance model generalization^30^. This dataset introduces enhanced real-world variability through:

#### Infrastructure diversity

Road surfaces characterized by irregular maintenance patterns, heterogeneous asphalt textures, and non-standardized or absent lane markings, commonly observed in rural and semi-urban regions.

#### Complex backgrounds

Images containing roadside vegetation, debris, surface stains, and variable shadow patterns, which strengthen the model’s ability to differentiate genuine pothole hazards from visually similar but non-critical surface irregularities. The inclusion of these complementary datasets enables the proposed model to learn domain-invariant and scale-robust features, thereby improving its generalization capability across diverse geographical regions and road environments. Although each dataset individually may appear moderate in size, the combined use of multi-source, multi-region datasets, along with controlled augmentation strategies, ensures sufficient diversity for training a robust pothole detection model. The inclusion of datasets from different countries, acquisition setups, and road environments enables the proposed model to learn domain-invariant features, thereby enhancing its generalization capability to unseen real-world scenarios. Sample images from all datasets are illustrated in Fig. 1.

### Training configuration and rationale

To ensure a fair and unbiased evaluation and to prevent any form of data leakage, all datasets were first randomly shuffled and then partitioned into mutually exclusive training, validation, and test sets prior to model training. A total of 9,862 images were utilized and divided using a 70:10:20 split for training, validation, and testing, respectively. The split was performed at the image level with stratification based on class labels, ensuring consistent class distribution across all subsets. Importantly, data augmentation was applied exclusively to the training set after the split. No augmented samples from the training data were included in the validation or test sets. The validation set was used solely for hyperparameter tuning and early stopping, while the test set was strictly held out and used only once for final performance evaluation. Furthermore, images originating from the same dataset source were randomly shuffled prior to splitting, and no images from the same scene or capture sequence were shared across different subsets. This strict separation protocol ensures that the reported performance metrics accurately reflect the generalization capability of the proposed model under unseen road conditions. The SegFormer-B4 model was trained using a learning rate of 0.001 for a maximum of 700 epochs, with an early stopping strategy employed based on validation performance to prevent overfitting. A batch size of 32 was selected to balance computational efficiency and gradient stability. Figure 4 shows the comparison of the training time in various models, i.e. YOLOv7, YOLOv8, Faster R-CNN, CC-ViT, and the proposed framework. It can be observed that YOLOv7 requires the highest training time (2.04 hours), followed by YOLOv8 (1.56 hours), Faster R-CNN (1.48 hours), and CC-ViT (1.34 hours), whereas the proposed model achieves the lowest training time of 1.07 hours. This training time is lower than that of the other two models and proves that the proposed framework is computationally efficient but with high detection performance.

### Statistical reliability of reported metrics

To ensure the reliability and reproducibility of the reported results, all experiments presented in Tables 2, 3, 4, 5 and 6 were conducted over five independent training runs using different random seeds while maintaining identical training, validation, and test splits for each dataset. The values of the reported precision, recall, mAP at 0.5, accuracy, and F1-score represent the average performance of these runs. Variance between runs was also low (usually in the range of ($\pm 0.5\%$ - 0.8%), and it showed the convergence of the proposed framework and comparative models. Since the trends were generally the same between several datasets and the variance between the results was not very high, the formal statistical significance testing was not reported separately. However, the repeated-run evaluation protocol ensures that the reported high performance values are robust and not attributable to random initialization effects.

### Depth estimation and severity representaion

Figure 5 displays the sample results of the proposed framework, in which the prediction of potholes is performed and their localization is achieved by identifying bounding boxes and the forecasted depth values of these boxes. The visual outputs can be used to show the extent to which the model is able to detect the location of potholes with relative precision to a variety of road conditions, including differences in road surface texture, luminance, and surface defects. The scores of confidence presented also show reliability of detection and depth estimation in varying conditions. The detected pothole bounding boxes along with their estimated depth values are illustrated in Fig. 5. As discussed in earlier sections, the depth and diameter parameters are used to estimate pothole severity levels, which directly support maintenance prioritization and safety assessment for road users. In addition to visual localization, the detection performance is quantitatively evaluated using Precision, Recall, Accuracy, F1-Score, and mAP@0.5, which are standard and widely accepted metrics for object detection and road damage assessment tasks. These metrics jointly capture localization accuracy, classification reliability, and detection completeness, thereby providing a comprehensive evaluation of model performance.

## Ablation study

An ablation study is conducted to systematically validate the contribution of each major component in the proposed framework. The architecture is incrementally constructed by integrating three key modules: (i) GAN-based image enhancement with SegFormer-B4, (ii) transfer learning applied to the SegFormer backbone, and (iii) fuzzy rule-based YOLOv9 integration. Figure 6 shows the performance of various architectural configurations as compared to several evaluation metrics such as Precision, Recall, mAP@0.5, Accuracy, and F1-Score. It can be observed that each successive integration of components leads to a consistent improvement in all metrics, with the final configuration (GAN+TL+SegFormer-B4+Fuzzy-YOLOv9) achieving the highest performance. The performance of each configuration is quantitatively evaluated using Precision, Recall, mAP@0.5, Accuracy, and F1-Score, as reported in Table 5 and visualized in Fig. 6. The use of multiple complementary metrics ensures that performance improvements are not confined to a single indicator, but reflect consistent gains in localization accuracy, detection completeness, and classification reliability. The results in Table 5 demonstrate a monotonic and substantial improvement across all evaluation metrics as each component is added to the framework. Incorporating transfer learning into the GAN-SegFormer-B4 baseline yields a clear performance gain, indicating improved feature adaptation and faster convergence. The subsequent integration of the fuzzy rule-based YOLOv9 module leads to a significant performance increase, particularly in accuracy (from 96.43% to 99.14%) and F1-score (from 93.54% to 97.82%). These improvements are consistent across all metrics, suggesting that the observed gains are systematic rather than incidental. Although formal hypothesis testing is not explicitly performed, the large effect size, consistent metric-wise improvements, and progressive performance gains across architectural stages provide strong empirical evidence of the statistical reliability of the proposed design. Furthermore, the robustness improvements indicate that fuzzy inference effectively mitigates ambiguity caused by shadows, reflections, and surface irregularities, thereby enhancing detection stability in real-world conditions.

**Table 5:** Ablation study performance comparison.

Models	Precision	Recall	mAP@0.5%	Accuracy	F1-Score
GAN with Segformer-B4	91.65	90.24	92.25	92.67	91.92
GAN+TL+Segformer-B4	95.87	92.58	94.62	96.43	93.54
GAN+TL+ Segformer-B4 +Fuzzy-Yolov9	98.79	97.81	99.23	99.14	97.82

### Comparative evaluation with strong baseline models

To ensure reliable performance comparison, the proposed framework is evaluated against state-of-the-art and widely adopted baseline models, including YOLOv7, YOLOv8, Faster R-CNN, and CC-ViT. These models represent both one-stage and two-stage detectors, as well as CNN-Transformer hybrid architectures, making them strong and diverse baselines for pothole detection

### Performance on Japan dataset

The Japan pothole dataset contains images captured under diverse real-world road conditions, including shadow occlusions, partial water accumulation, reflective glare, and uneven illumination, as observed from the dataset repository. The proposed model’s performance on the Japan dataset, referred to as the Potholes Japan Computer Vision Project, is superior to the other models, achieving an accuracy of 98.92%, mAP of 97.82%, recall of 96.21%, F1-score of 98.14%, and precision of 97.84%. Unlike baseline models that exhibit trade-offs between recall and precision, the proposed framework maintains consistently high values across all evaluation metrics, indicating balanced detection performance. A comparative study of the various models on the Japan dataset in important evaluation metrics is given in Fig. 7 in Precision, Recall, mAP@0.5, Accuracy, and F1-Score. It is possible to see that the proposed model has the highest values of all metrics, which gives evidence to the excellent performance of the detection of baselines and well-balanced performance. The CC-ViT model also demonstrates competitive performance with an accuracy of 96.85%, mAP of 96.34%, recall of 95.14%, and precision of 95.87%. Faster-RCNN delivers moderate results on this dataset, whereas YOLOv8 and YOLOv7 produce comparatively lower values across all performance metrics. The obtained results are summarized in Table 2 and illustrated in Fig. 7.

### Performance on POTHOLEV3 dataset

As shown in Fig. 8, the various models perform comparatively on the POTHOLEV3 dataset with the various evaluation measures being Precision, Recall, mAP@0.5, Accuracy and F1-score. It can be observed that there is an upward trend in the consistency of the improvement between YOLOv7 and the proposed model, as the values are maximum in all metrics, which implies strong detection performance in challenging road setups. The experimental results obtained on the POTHOLEV3 dataset are presented in Table 3 and illustrated in Fig. 8. Among the comparative models, the CC-ViT model achieves performance values close to the proposed approach, with an accuracy of 97.54% and recall of 96.13%, compared to the proposed model’s 98.28% accuracy and 97.21% recall. The relatively smaller variation among Faster-RCNN, CC-ViT, and the proposed model can be attributed to their hybrid architectural designs, whereas YOLOv7 and YOLOv8 exhibit comparatively lower performance due to their simpler detection structures. Nevertheless, the proposed model consistently outperforms all other approaches on the POTHOLEV3 dataset, achieving an accuracy of 98.28%, recall of 97.21%, precision of 98.58%, F1-score of 98.41%, and mAP@0.5 of 98.59%, respectively. These results confirm the superior detection capability and generalization strength of the proposed framework on complex road surface conditions represented in the POTHOLEV3 dataset.The consistent performance gains across both Japan and POTHOLEV3 datasets confirm that the proposed model does not overfit to a specific dataset and exhibits strong generalization capability under varied road and illumination conditions.

### Performance on pothole detection dataset-v2-2022

Figure 9 shows the relative performance of various models on the Pothole Detection Dataset-v2-2022 on the most important metrics, such as Precision, Recall, mAP@0.5, Accuracy and F1-score. As the figure reveals, there is a relatively high level of performance in all models on this dataset as the quality of images and resolution are quite high and the proposed model consistently obtains the highest values in all metrics. The Pothole Detection Dataset-v2-2022 is a collection of pothole images which are of high quality and high-resolution. Therefore, all tested models perform excellently on this dataset than the earlier two datasets. As it is observed in Table 6, each of the models has its highest performance in this dataset, which is indicative that the image quality, resolution, and camera angle are also significant factors in predicting the accuracy of pothole detection. Despite the improvement of the overall performance of all methods, the proposed model has always taken the first place in the comparative approaches regarding the accuracy of 98.79, mAP at 0.5 of 98.18, recall of 97.86, F1-score of 97.42, and precision of 98.64. It is also demonstrated that the performance of the CC-ViT and Faster-RCNN models is also competitive, though, their values are lower than the values of the proposed framework. In Fig. 9, one can see the comparative performance of all models on the Pothole Detection Dataset-v2-2022 in terms of several evaluation metrics. The superior performance under identical evaluation conditions further validates the reliability of the proposed approach when compared against strong baseline detectors.

### Ablation analysis: impact of GAN-based pre-processing

To explicitly evaluate the contribution of the GAN-based pre-processing stage, an ablation study was conducted by training and testing the proposed framework with and without GAN-based image enhancement, while keeping all other components SegFormer-B4, YOLOv9, fuzzy inference rules, training configuration, and datasets unchanged. This controlled comparison isolates the effect of GAN pre-processing on pothole detection performance.

**Table 6:** Ablation analysis of GAN-based pre-processing.

Dataset	GAN	Precision (%)	Recall (%)	mAP@0.5 (%)	Accuracy (%)	F1-score (%)
Japan	No GAN	95.91	93.84	94.12	96.47	94.86
Japan	With GAN	**97.84**	**96.21**	**97.82**	**98.92**	**98.14**
POTHOLEV3	No GAN	96.42	94.65	95.71	96.89	95.52
POTHOLEV3	With GAN	**98.58**	**97.21**	**98.59**	**98.28**	**98.41**
PD-v2-2022	No GAN	97.31	96.04	96.85	97.42	96.68
PD-v2-2022	With GAN	**98.64**	**97.86**	**98.18**	**98.79**	**97.42**

**Figure 10:**
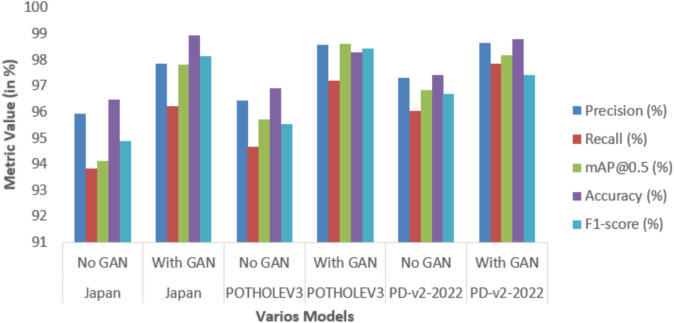
Ablation analysis of GAN-based preprocessing across three datasets (Japan, POTHOLEV3, and pothole detection dataset-v2-2022).

A comparison of model performance with and without GAN-based preprocessing in three datasets (Japan, POTHOLEV3 and PD-v2-2022) is shown in Fig. 10. As presented in the figure, it is evident that the use of GAN enhancement has a consistent positive impact on all the evaluation measures, such as Precision, Recall, mAP 0.5, Accuracy, and F1-score, in various datasets. The difference in performance in terms of works done by GAN versus non-GAN demonstrates the success of GAN in improving feature quality and detectability. Table 6 and Fig. 10 shows the obtained results (compared) on Japan, POTHOLEV3 and Pothole Detection Dataset-v2-2022 datasets. With the removal of GAN preprocessing, a steady drop in performance is witnessed across all the evaluation measures, especially the ones related to recall, mAP@0.5 and F1-score. The decrease in performance is greater on the datasets involving difficult scenarios like shadows, potholes full of water, and diffused lighting (Japan and POTHOLEV3), which suggests that GAN-based enhancement is a key component in enhancing features and boundary separability in unfavourable visual conditions.

The inclusion of GAN preprocessing improves contrast normalization and enhances pothole boundary definition, which directly benefits the metric-aware segmentation and geometric parameter extraction stages. As a result, downstream severity estimation using fuzzy inference becomes more reliable. These findings confirm that the GAN module is not merely a cosmetic preprocessing step, but a functionally significant component that contributes to improved robustness and generalization of the proposed framework across diverse real-world scenarios.

### Real-time performance analysis and computational feasibility

In addition to accuracy-based metrics, real-time performance and computational feasibility were analyzed to provide a holistic evaluation. The proposed framework achieves lower inference time than Faster R-CNN and CC-ViT while maintaining superior detection accuracy, demonstrating an effective accuracy–efficiency trade-off. The computational efficiency of the suggested structure was considered in regard to the inference speed, latency and hardware demands. Since YOLOv9 is primarily designed for real-time applications, the proposed framework was further evaluated for computational efficiency. The test was conducted using an NVIDIA RTX 3080 graphics card, Intel i9 processor and 32 GB RAM. The proposed framework exhibited a much lower inference time as compared to Faster-RCNN and CC-ViT, but at the same time, the model showed a better detection rate. The fuzzy inference block had minimal computing requirements, and the real time efficiency was maintained. This evidence suggests that the suggested architecture will provide a good tradeoff between the accuracy of detection and computation costs.

### Robustness under challenging real-world conditions

Pothole images that were subjected to shadows, water-filled surface, and glare reflection were visually viewed and studied across the three publicly available datasets (Japan, POTHOLEV3, and Pothole Detection Dataset-v2-2022). The suggested structure was able to sustain constant performance of detection in these harsh conditions. Featuring a fuzzy inference layer that optimally overcomes uncertainty caused by reflective surfaces, partial occlusion, and ambiguity at boundary, and featuring stable and precise localization with YOLOv9, these layers are compatible. The contrast in shadow-affected areas is additionally increased by the GAN-based preprocessing and leads to the increase in the detection reliability. This strength proves that the suggested framework is capable of functioning under realistic conditions of road inspection. Qualitative evaluations under shadow, water-filled surfaces, glare, and partial occlusion further confirm the robustness of the proposed framework. The combined effect of GAN-based preprocessing, fuzzy inference, and YOLOv9 detection ensures stable performance under realistic road inspection scenarios.

### Discussion on findings

In our proposed approach, the integration of GAN generator and Discriminator has effectively enhanced the detection accuracy along with transfer learning methods, especially Fuzzy Based Rules played a crucial role in detecting potholes from the input dataset images. In literature, the existing works were not focused on the combination of SegFormer4 and Fuzzy based rules together along with GAN model and YOLOv9. From the ablation study of this research paper, it is evident that each module of this hybrid approach has significantly enhanced the results of pothole detection accuracy and other evaluation metric, specifically after the integration of SegFormer4 and Fuzzy Based rules which is given in Table 3. Moreover, we have considered both diameter and depth of the pothole for estimating the original values, thus the severity of such potholes are determined and respective recommendations were suggested. Therefore, these results on different datasets implies that the proposed approach have significant advantage over the other state of the art approaches considered in this study in terms of various performance evaluation metrics.

## Conclusion and future work

Ensuring road safety is a crucial objective for every country to protect human lives and support economic growth through efficient transportation systems. Although traditional road inspection methods were effective in earlier years, the increasing complexity of road networks and traffic conditions has necessitated the adoption of deep learning-based approaches to improve pothole detection accuracy and reliability. In this paper, a hybrid framework combining a GAN model with a realistic discriminator for image enhancement, transfer learning-based SegFormer-B4 for segmentation, and a fuzzy-integrated YOLOv9 model for severity classification has been proposed. The suggested system estimates the severity of the potholes, based on the diameter and depth characteristics, and recommends on changes in maintenance. The experimental findings on the datasets of Japan, POTHOLEV3, and Pothole Detection Dataset-v2-2022 evidence that the proposed framework has better detection accuracy regarding precision, recall, accuracy, and mAP at 0.5. The stability of the proposed model can also be demonstrated by its ability to perform smoothly in varying illumination conditions, texture of the surface, and other factors in the environment in various datasets. The fuzzy inference system also increases reliability through the depth estimation uncertainty and boundary ambiguity. Even though the proposed framework is capable of implementing real-time on the hardware of GPUs, it requires the presence of specific computational resources. Transformer-based components and segmentation backbones increase memory consumption compared to lightweight CNN architectures. Nevertheless, the combination with YOLOv9 makes the inference much less complex than two-stage detectors. Nevertheless, there are still some constraints. Approximation of the depth and diameter is guided by image-based estimations, which are not validated physically, and the bias in the datasets toward certain areas can have an impact on the transfer to unknown road conditions. Secondly, the field deployment validation has not been carried out on a large scale yet. Future directions will be to integrate improved transfer learning strategies and enhanced YOLO architecture with advanced features in crack detection and depth estimation to be verified with physical measurement sensors. In addition, next generation research will involve the use of bigger multi-country data and real time field implementation to enhance strength and generalization further. On the whole, the suggested framework manages to utilize the advantages of GAN, SegFormer-B4, and fuzzy-YOLOv9 to provide high dependability of potholes in the form of detection and classification of severity, which helps to make road infrastructure management safer and more sustainable.

## Data Availability

1. Japan Dataset: https://universe.roboflow.com/nikosworkspace/potholes-japan/dataset/3. 2. POTHOLEV3 Dataset: https://universe.roboflow.com/pothole-detection-y1mqk/pothole-detection-i09mh/model/3. 3. Pothole Detection Dataset-v2-2022: https://www.kaggle.com/datasets/rajdalsaniya/pothole-detection-dataset.

## References

[1] Yu, J. et al. Road surface defect detection—from image-based to non-image-based: a survey. *IEEE Trans. Intell. Transp. Syst.* (2024).

[2] Singh, S., Chhabra, R. & Gill, R. An empirical review of potholes classification using road images. *Manuf. Technol. Prod. Syst.* 171–180 (2023).

[3] Kothai, R., Prabakaran, N., Murthy, Y. S., Cenkeramaddi, L. R. & Kakani, V. Pavement distress detection, classification and analysis using machine learning algorithms: a survey. *IEEE Access* (2024).

[4] Tripathy, A., Rajalakshmi, T., Suryakala, S. V. et al. Image-based pothole detection system using yolov8 algorithm. In *2024 International Conference on Recent Advances in Electrical, Electronics, Ubiquitous Communication, and Computational Intelligence (RAEEUCCI)*, 1–5 (IEEE) (2024).

[5] Matouq, Y., Manasreh, D. & Nazzal, M. D. Ai-driven approach for automated real-time pothole detection, localization, and area estimation. *Transp. Res. Rec.* **2678**, 2018–2031 (2024).

[6] Kim, Y.-M. et al. Review of recent automated pothole-detection methods. *Appl. Sci.* **12**, 5320 (2022).

[7] Park, S.-S., Tran, V.-T. & Lee, D.-E. Application of various yolo models for computer vision-based real-time pothole detection. *Appl. Sci.* **11**, 11229 (2021).

[8] Fan, R. & Liu, M. Road damage detection based on unsupervised disparity map segmentation. *IEEE Trans. Intell. Transp. Syst.* **21**, 4906–4911 (2019).

[9] Koch, C. & Brilakis, I. Pothole detection in asphalt pavement images. *Adv. Eng. Inform.* **25**, 507–515 (2011).

[10] Gomes Correia, M. & Ferreira, A. Road asset management and the vehicles of the future: an overview, opportunities, and challenges. *Int. J. Intell. Transp. Syst. Res.* **21**, 376–393 (2023).

[11] Manasreh, D. et al. Application of autonomous vehicles for automated roadside safety assessment. *Transp. Res. Rec.* **2676**, 255–266 (2022).

[12] Biçici, S. & Zeybek, M. An approach for the automated extraction of road surface distress from a uav-derived point cloud. *Autom. Constr.* **122**, 103475 (2021).

[13] Ravi, R., Bullock, D. & Habib, A. Pavement distress and debris detection using a mobile mapping system with 2d profiler lidar. *Transp. Res. Rec.* **2675**, 428–438 (2021).

[14] Loganathan, K. et al. Estimated remaining fatigue life of flexible pavements based on the normalized comprehensive area ratio deflection parameter. *Can. J. Civ. Eng.* **47**, 546–555 (2020).

[15] Benmhahe, B. & Chentoufi, J. A. Automated pavement distress detection, classification and measurement: A review. *Int. J. Adv. Comput. Sci. Appl.* **12** (2021).

[16] Ghosh, R. & Smadi, O. Automated detection and classification of pavement distresses using 3d pavement surface images and deep learning. *Transp. Res. Rec.* **2675**, 1359–1374 (2021).

[17] Zhang, A. A. et al. Intelligent pavement condition survey: overview of current researches and practices. *J. Road Eng.* (2024).

[18] Roman-Garay, M. et al. Architecture for pavement pothole evaluation using deep learning, machine vision, and fuzzy logic. *Case Stud. Constr. Mater.* **22**, e04440 (2025).

[19] Kothai, R. & Prabakaran, N. Self attention gan and swin transformer based pothole detection with trust region based lsm and hough line transform for 2d to 3d conversion. *IEEE Access* (2025).

[20] Satti, S. K., Rajareddy, G. N., Mishra, K. & Gandomi, A. H. Potholes and traffic signs detection by classifier with vision transformers. *Sci. Rep.* **14**, 2215 (2024). 38278836 10.1038/s41598-024-52426-4 PMC10817940

[21] Katsamenis, I. et al. Deep transformer networks for precise pothole segmentation tasks. In *Proceedings of the 16th International Conference on PErvasive Technologies Related to Assistive Environments*, 596–602 (2023).

[22] Talha, S. A., Manasreh, D. & Nazzal, M. D. The use of lidar and artificial intelligence algorithms for detection and size estimation of potholes. *Buildings* **14**, 1078 (2024).

[23] Heo, D.-H., Choi, J.-Y., Kim, S.-B., Tak, T.-O. & Zhang, S.-P. Image-based pothole detection using multi-scale feature network and risk assessment. *Electronics* **12**, 826 (2023).

[24] Lincy, A., Dhanarajan, G., Kumar, S. S. & Gobinath, B. Road pothole detection system. In *ITM Web of Conferences*, **53**, 01008 (EDP Sciences)(2023).

[25] Wang, N., Shang, L. & Song, X. A transformer-optimized deep learning network for road damage detection and tracking. *Sensors* **23**, 7395 (2023). 37687850 10.3390/s23177395 PMC10490637

[26] Fan, R., Wang, H., Bocus, M. J. & Liu, M. We learn better road pothole detection: from attention aggregation to adversarial domain adaptation. In *European conference on computer vision*, 285–300 (Springer)(2020).

[27] Ranieri, A., Thompson, E. M. & Biasotti, S. Automatic structural health monitoring of road surfaces using artificial intelligence and deep learning. In *Data Driven Methods for Civil Structural Health Monitoring and Resilience*, 297–311 (CRC Press)(2023).

[28] Safyari, Y., Mahdianpari, M. & Shiri, H. A review of vision-based pothole detection methods using computer vision and machine learning. *Sensors* **24**, 5652 (2024). 39275561 10.3390/s24175652 PMC11397941

[29] Xu, M., Yoon, S., Fuentes, A. & Park, D. S. A comprehensive survey of image augmentation techniques for deep learning. *Pattern Recogn.* **137**, 109347 (2023).

[30] Ma, N. et al. Computer vision for road imaging and pothole detection: a state-of-the-art review of systems and algorithms. *Transp. Saf. Environ.* **4**, tdac026 (2022).

